# Curcumin-mediated transcriptional regulation of human *N*-acetylgalactosamine-α2,6-sialyltransferase which synthesizes sialyl-Tn antigen in HCT116 human colon cancer cells

**DOI:** 10.3389/fmolb.2022.985648

**Published:** 2022-09-12

**Authors:** So-Young An, Kyoung-Sook Kim, Jong-Hyun Cho, Hee-Do Kim, Cheorl-Ho Kim, Young-Choon Lee

**Affiliations:** ^1^ Department of Medicinal Biotechnology, College of Health Sciences, Dong-A University, Busan, South Korea; ^2^ Molecular and Cellular Glycobiology Unit, Department of Biological Sciences, SungKyunKwan University, Kyunggi-Do, South Korea

**Keywords:** curcumin, human sialyltransferase (hST6GalNAc I), human colon carcinoma, estrogen receptor α binding site, transcriptional activation

## Abstract

Human *N*-acetylgalactosamine-α2,6-sialyltransferase (hST6GalNAc I) is the major enzyme involved in the biosynthesis of sialyl-Tn antigen (sTn), which is known to be expressed in more than 80% of human carcinomas and correlated with poor prognosis in cancer patients. Athough high expression of hST6GalNAc I is associated with augmented proliferation, migration and invasion in various cancer cells, transcriptional mechanism regulating hST6GalNAc I gene expression remains largely unknown. In this study, we found that hST6GalNAc I gene expression was markedly augmented by curcumin in HCT116 human colon carcinoma cells. To understand the molecular mechanism for the upregulation of hST6GalNAc I gene expression by curcumin in HCT116 cells, we first determined the transcriptional start site of hST6GalNAc I gene by 5′-RACE and cloned the proximal hST6GalNAc I 5′-flanking region spanning about 2 kb by PCR. Functional analysis of the hST6GalNAc I 5′ flanking region of hST6GalNAc I by sequential 5′-deletion, transient transfection of reporter gene constructs and luciferase reporter assays showed that -378/-136 region is essential for maximal activation of transcription in response to curcumin in HCT 116 cells. This region includes putative binding sites for transcription factors c-Ets-1, NF-1, GATA-1, ER-α, YY1, and GR-α. ChIP analysis and site-directed mutagenesis demonstrated that estrogen receptor α (ER-α) binding site (nucleotides -248/-238) in this region is crucial for hST6GalNAc I gene transcription in response to curcumin stimulation in HCT116 cells. The transcription activity of hST6GalNAc I gene induced by curcumin in HCT116 cells was strongly inhibited by PKC inhibitor (Gö6983) and ERK inhibitor (U0126). These results suggest that curcumin-induced hST6GalNAc I gene expression in HCT116 cells is modulated through PKC/ERKs signal pathway.

## Introduction

Sialyl-Tn (sTn) antigen, a simple mucin-type carbohydrate antigen, is rarely detected in normal adult tissues, while it is plentifully expressed in more than 80% of human carcinomas, including colorectal (Itzkowitz et al., 1990), breast (Leivonen et al., 2001), ovarian (Kobayashi et al., 1992), pancreatic (Itzkowitz et al., 1991; Kim et al., 2002), gastric (Victorzon et al., 1996; Baldus and Hanisch, 2000), and endometrial (Hashiguchi et al., 2016) carcinomas, in which it is closely associated with tumorigenesis, metastasis, chemoresistance, immune suppression and poor prognosis (Julien et al., 2012; Munkley, 2016).

Human sTn antigen is synthesized by human *N*-acetylgalactosamine-α2,6-sialyltransferase (hST6GalNAc I) which catalyzes the addition of sialic acid (Sia) in an α2,6-linkage to GalNAc α-O-Serine/Threonine (Ser/Thr), resulting in the formation of sTn antigen (Siaα2-6GalNAcα-O-Ser/Thr) structure in the O-linked carbohydrate chains of glycoprotein (Sewell et al., 2006; Julien et al., 2012; Munkley, 2016). Previous studies have reported the augmented mRNA expression of hST6GalNAc I in serum samples and gastrointestinal tissues of patients with gastric cancer (Marcos et al., 2003, 2011; Bhat et al., 2018). High expression of hST6GalNAc I has also been observed in breast, prostate and ovarian cancer cell lines displaying elevated level of sTn antigen, which directly correlates with enhanced metastasis and tumorigenicity (Sewell et al., 2006; Munkley et al., 2015; Wang et al., 2019). In addition, silencing of hST6GalNAc I gene using RNA interference (RNAi) technology decreased sTn antigen expression level and simultaneously inhibited cell growth, migration, and invasion of gastric cancer and hepatocarcinoma cells (Tamura et al., 2016; Yu et al., 2021).

It has been proven that human sialyltransferases are expressed in a tissue- and cell type-specific manner and their expressions are tightly regulated at the transcriptional level under the control of specific promoters (Dall’Olio and Chiricolo, 2001; Harduin-Lepers et al., 2001; Dorsett et al., 2021). In order to understand the molecular mechanism for overexpression of hSTGalNAc I in cancer cells, it is crucial to elucidate the transcriptional regulation of the hST6GalNAc I gene in cancer cells. A previous study by Munkley et al. (2015) has shown that expresson of hST6GalNAc1 gene is upregulated significantly in primary prostate carcinoma but downregulated in established metastatic tissue (Munkley et al., 2015). Moreover, they demonstrated that expressions of hST6GalNAc1 gene and sTn is induced by androgen in human prostate cancer cell line LNCaP, and confirmed using ChIP-qPCR that androgen-inducible hST6GalNAc1 gene expression in LNCaP cells is conducted by direct binding of androgen to an androgen receptor-binding site in close proximity to the hST6GalNAc1 promoter (Munkley et al., 2015). It was reported that transcript levels of hST6GalNAc I was time-dependently increased with an enhancement of the homeobox transcription factor CDX2 protein expression during differentiation of Caco-2 intestinal cell line (Pinto et al., 2015). In addition, ChIP analysis demonstrated that CDX2 binds to the regulatory region of hST8GalNAc I gene in Caco-2 cells and human intestinal metaplasia and luciferase reporter assay showed that CDX2 transactivates the regulatory region of hST8GalNAc I gene in human gastric carcinoma cell lines AGS and MKN45 (Pinto et al., 2015).

In recent decade, we have studied the specific gene expression of human sialyltransferases mediating formation of the sialylated glycans of glycoproteins and glycolipids by natural compounds (Kwon et al., 2010; Baik et al., 2014; Yoon et al., 2016; Lee et al., 2018a; 2018b). In this study, we have found the marked increase of hST6GalNAc I gene expression by curcumin in HCT116 human colon carcinoma cells. To understand the molecular basis of hST6GalNAc I gene expression triggered by curcumin, furthermore, we characterized the curcumin-responsive promoter region to mediate the upregulation of hST6GalNAc I gene expression in HCT116 cells.

## Materials and methods

### Cell culture

Human colon cancer cell line HCT116, lung cancer cell line A549, glioblastoma cell line U-87 MG and breast cancer cell line MCF-7 obtained from Korean Cell Line Bank (KCLB, Seoul, Korea) were cultivated and maintained in Dulbecco’s modifed Eagle’s medium (DMEM; WelGENE Co., Korea) containing 10% (v/v) heat-inactivated fetal bovine serum (FBS) and 1% PSA (WelGENE Co., Korea) as previously described (Lee et al., 2018a; 2018b). Curcumin (Sigma-Aldrich) was dissolved in dimethyl sulfoxide (DMSO) at 10 mM for stock concentration, and stored at -20°C.

### Reverse transcription-polymerase chain reaction

Total RNA was extracted from cells treated with various concentrations of curcumin for 24 h using Trizol reagent (Invitrogen). RT-PCR was performed using gene-specific primers (Table 1) as previously described (Lee et al., 2018a, 2018b; An et al., 2020). PCR products were analyzed by 1.5% agarose gel electrophoresis and visualized with ethidium bromide. The intensity of the amplified DNA band was measured with a Scion Image Instrument (Scion Corp.; Frederick, MD, United States).

**TABLE 1:** Primer sequences used in this study.

Primer	Sequence	Strand	Purpose
hST6GalNAc I	5′-TAT​CGT​AAG​CTG​CAC​CCC​AAT​C-3′	Sense	RT-PCR
hST6GalNAc I	5′-TTA​GCA​GTG​AAT​GGT​CCG​GAA​G-3′	Antisense	RT-PCR
β-actin	5′-CAA​GAG​ATG​GCC​ACG​GCT​GCT-3′	Sense	RT-PCR
β-actin	5′-TCC​TTC​TGC​ATC​CTG​TCG​GCA-3′	Antisense	RT-PCR
GSP-RT	5′-CTG​GCT​CTT​CCA​TGA​TTG-3′	Antisense	5′-RACE
GSP1	5′-GCC​ATC​CCT​GCA​TCT​TGC​CCT​CTG​G-3′	Antisense	5′-RACE
GSP2	5′-TCC​ATG​CTG​CCC​TCT​GTG​CTG​TGT​GG-3′	Antisense	5′-RACE
P-1974S	5′-ATGGT​ACCGAT​TGT​CCA​CAG​TAG​CTC​AGC​TTG​C-3′	Sense	Deletion
P-1575S	5′-ATGGT​ACCCTT​GCC​ACC​ATG​ACT​TGA​AAC​AGT​GC-3′	Sense	Deletion
P-1118S	5′-ATGGT​ACCCTC​GTA​GGT​GCT​ACT​AAG​CAG​TGG-3′	Sense	Deletion
P-749S	5′-ATGGT​ACCGTA​ATC​CCA​GCA​CTT​TGG​GAG​GC-3′	Sense	Deletion
P-378S	5′-ATGGT​ACCGTG​TTG​AAC​ATG​TGT​GTT​GGG​TGC​C-3′	Sense	Deletion
P-136S	5′-ATGGT​ACCGGA​GTT​TCC​CTT​CCT​TTA​AGC​CAC​G-3′	Sense	Deletion
P+1A	5′-GTCTC​GAGGTC​ACA​CCC​TTT​GTC​TTA​ACA​ATG​AGC​C-3′	Antisense	Deletion
GR-α mut	5′-GTT​GAG​CTA​AAG​TGT​TGA ***CGC*** TGT​GTG​TTG​GGT​GCC​TAC​C-3′	Sense	Mutagenesis
GR-α mut	5′-GGT​AGG​CAC​CCA​ACA​CAC​A ***GCG*** TCA​ACA​CTT​TAG​CTC​AAC-3′	Antisense	Mutagenesis
c-Ets-1(-294) mut	5′-CTT​CAT​GAT​AAT​CCC​AAA​A ***GGC​C*** GAT​AAC​TGC​TTT​TTT​CC-3′	Sense	Mutagenesis
c-Ets-1(-294) mut	5′-GGA​AAA​AAG​CAG​TTA​TC ***GGC​C*** TTT​TGG​GAT​TAT​CAT​GAA​G-3′	Antisense	Mutagenesis
GATA-1(-290) mut	5′-CTT​CAT​GAT​AAT​CCC​AAA​AGG​AA ***GGC​C*** ACT​GCT​TTT​TTC​C-3′	Sense	Mutagenesis
GATA-1(-290) mut	5′-GGA​AAA​AAG​CAG​T ***GGC​C*** TTC​CTT​TTG​GGA​TTA​TCA​TGA​AG-3′	Antisense	Mutagenesis
YY1 mut	5′-GCT​TTT​TTC​CAT​TTA​T ***CCG​G*** CGG​CAG​ATG​GGT​TAA​G-3′	Sense	Mutagenesis
YY1 mut	5′-CTT​AAC​CCA​TCT​GCC​G ***CCG​G*** ATA​AAT​GGA​AAA​AAG​C-3′	Antisense	Mutagenesis
ER-α mut	5′-GAT​GGG​TTA​AGT​AAC​C ***GGG​C*** CAA​GAT​GAG​ACT​GC-3′	Sense	Mutagenesis
ER-α mut	5′-GCA​GTC​TCA​TCT​TG ***GCC​C*** GGT​TAC​TTA​ACC​CAT​C-3′	Antisense	Mutagenesis
GATA-1(-185) mut	5′-CAT​AAA​GCT​TGA​CTT​TCA ***GGC​C*** TTG​GCT​TCA​AAG​ACA​AAA​AAG​G-3′	Sense	Mutagenesis
GATA-1(-185) mut	5′-CCT​TTT​TTG​TCT​TTG​AAG​CCA​A ***GGC​C*** TGA​AAG​TCA​AGC​TTT​ATG-3′	Antisense	Mutagenesis
C/EBPβ mut	5′-GCT​TGA​CTT​TCA​GAT​A ***GGC​C*** CTT​CAA​AGA​CAA​AAA​AGG​AAG​G-3′	Sense	Mutagenesis
C/EBPβ mut	5′-CCT​TCC​TTT​TTT​GTC​TTT​GAA​G ***GGC​C*** TAT​CTG​AAA​GTC​AAG​C-3′	Antisense	Mutagenesis
c-Ets-1(-160) mut	5′-GGC​TTC​AAA​GAC​AAA​AAA ***GGC​C*** GGT​AAA​CAT​GTT​TGA​AC-3′	Sense	Mutagenesis
c-Ets-1(-160) mut	5′-GTT​CAA​ACA​TGT​TTA​CC ***GGC​C*** TTT​TTT​GTC​TTT​GAA​GCC-3′	Antisense	Mutagenesis
ER-α (195 bp)	5′-GCA​TGG​TGG​CCT​CAA​GG-3′	Sense	ChIP
ER-α (195 bp)	5′-GTG​TTC​AAA​CAT​GTT​TAC​CTT​CC-3′	Antisense	ChIP
ER-α (475 bp)	5′-AAC​ACA​AGC​GCC​AGA​GAG​ATG-3′	Sense	RT-PCR
ER-α (475 bp)	5′-GAT​CTC​CAC​CAT​GCC​CTC​TAC-3′	Antisense	RT-PCR

Primers P-1974S to P+1A were used for the isolation of 5′-flanking region of the hST6GalNAc I gene and for the construction of the deletion mutants. These contain *Kpn*I and *Xho*I sites underlined in sense and antisense primers, respectively. The mutated nucleotides in the oligonucleotides for mutation are in boldface and italic type.

### 5′-amplification of cDNA ends

Total RNA from HCT116 cells treated with 50 μM curcumin for 24 h was extracted using Trizol reagent (Invitrogen). Amplification of the 5′-end of hST6GalNAc I was conducted with gene-specific primers (Table 1) and the GeneRacer kit (Invitrogen, United States) according to the manufacturer’s instructions, as previously described (Lee et al., 2018b). The amplified product was analyzed on a 1% (w/v) agarose gel and purified using GeneAll Expin Gel SV kit (GeneAll biotechnology, Korea). The purified PCR product was subcloned into pGEM-T Easy vector (Promega, United States) and sequenced by Macrogen Company (Seoul, South Korea).

### Isolation of the 5′-Flanking region of the hST6GalNAc I gene

We isolated the 5′-flanking region of the hST6GalNAc I gene by PCR using the sequence information of the National Center for Biotechnology Information (NCBI Reference Sequence NC_000017.11). This was conducted in a similar manner to the experimental method described previously (An et al., 2020). A 1974 bp fragment of the 5′-flanking sequence of the hST6GalNAc I gene were amplified by long and accurate PCR (LA-PCR) using human genomic DNA (Clontech) as template, and a sense primer P-1974S and an antisense primer P + 1A containing *Kpn*I and *Xho*I sites, respectively (Table 1). LA-PCR condition was as follows: 95 °C for 5 min, then 30 cycles of 95 °C for 40 s, 55°C for 40s, 72°C for 45s and finally 72°C for 7 min. The 1974 bp PCR product was gel-purified, subcloned into pGEM-T Easy vector, resulting in pGEM-hST6GalNAc I, and sequenced, as described above.

### Construction of luciferase reporter plasmids

The *Kpn*I/*Xho*I-digested fragment (1974 bp) of the pGEM-hST6GalNAc I was subcloned into the *Kpn*I/*Xho*I site of the reporter plasmid, pGL3-Basic vector (Promega), to be fused to the luciferase gene (pGL3-1974). For the functional characterization of the hST6GalNAc I promoter, five different constructs (pGL3-136 to pGL3-1375) with 5′- deletion of hST6GalNAc I promoter were generated by LA-PCR amplification using pGEM-hST6GalNAc I as template with sense and antisense primers containing *Kpn*I and *Xho*I sites, respectively (Table 1). The amplified DNA fragments were subcloned into pGEM-T Easy vector. Then, they were digested with *Kpn*I and *Xho*I and introduced into the corresponding sites of the pGL3- Basic vector. The sequence and correct orientation of each deletion construct were verified by restriction analysis and sequencing.

### Transient cell transfection and luciferase reporter assays

We performed transfection and luciferase assay following the same procedure used previously (Lee et al., 2018a; 2018b). Briefly, cells were grown in DMEM containing 10% FBS until the cells were about 70% confluent in 24-well plates. The transfection complex containing luciferase reporter plasmid (0.5 µg), pRL-TK plasmid (50 ng) as an internal control, Lipofectamine^TM^ transfection reagent (Invitrogen), and serum-free Opti-MEM (Gibco-BRL) was added to each well and incubated for 8 h. Then, after removal of the transfection comlex, the cells were cultured in DMEM containing 10% FBS and 50 µM curcumin for 24 h. Then, cells were harvested and lysed with Passive Lysis Buffer (Promega). Luciferase assay was performed using the Dual-Luciferase Reporter Assay System (Promega) with a GloMax^TM^ 20/20 luminometer (Promega). Transfection efficiencies were normalized to *Renilla* luciferase activity of the pRL-TK vector. Experiments were carried out in triplicate. The data are presented as means ± SEM.

### Bioinformatics analysis

*In silico* analysis were performed with the ALGGEN-PROMO.v8.3 online software (http://alggen.lsi.upc.es/cgibin/promo_v3/promo/promoinit.cgi?dirDB=TF_8.3) to identify the putative transcription factor binding sites in the DNA sequences of the hST6GalNAc I promoter. An 100% matrix similarity rate was applied.

### Site-directed mutagenesis

Mutations with base substitution in the pGL3-378 construct were generated using a EZchange^TM^ Site-directed Mutagenesis kit (Enzynomics, Korea) according to the manufacturer’s protocol using oligonucleotide primers (Table 1). The desired mutations were verified by DNA sequencing.

### Chromatin immunoprecipitation assay

ChIP assay was conducted to assess the interaction between ERα and ERE located in hST6GalNAc I promoter using the ChIP kit (Upstate Biotechonology) according to the same protocol described previously (Lee et al., 2018a, 2018b; An et al., 2020). Immunoprecipitation was performed using 5 µg of anti-ERα antibody (ab32063, Abcam) and IgG antibodies (Sigma). The precipitated DNA was amplified by PCR for fragments of estrogen response element (ERE) in hST6GalNAc I promoter. Primers for ChIP assay are listed in Table 1.

### Flow cytometry analysis

For sTn staining, 1 × 10^6^ cells were incubated in 250 μl of BD Cytofix/Cytoperm solution (BD Biosciences, United States) for 20 min at room temperature. After washing three times with Dulbecco’s phosphate-buffered saline (DPBS), the cells were incubated with shaking overnight at 4°C in 300 μl of PBS containing 1% BSA with 1 μg of the anti-sialyl Tn antibody (Abcam, ab115957). After centrifugation at 3000 rpm for 2 min, the cells were were resuspended in 300 μl of PBS containing 1% BSA with Alexa Fluor 488-conjugated goat anti-mouse IgG H&L secondary antibody (1:2000; ab150113; Abacm) and incubated in the dark for 30 min at room temperature. The stained cells were washed three times with DPBS, resuspended with 500 μl of DPBS, and analyzed using the Attune NxT Acoustic Focusing Cytometer (Thermo Fisher Scientific).

### Statistical analysis

Results are expressed as mean ± SEM. Data were analyzed by Student’s t-test. *p* < 0.05 was considered statistically significant.

## Results

### Effect of curcumin on hST6GalNAc I gene expression in human cancer cell lines

**FIGURE 1:**
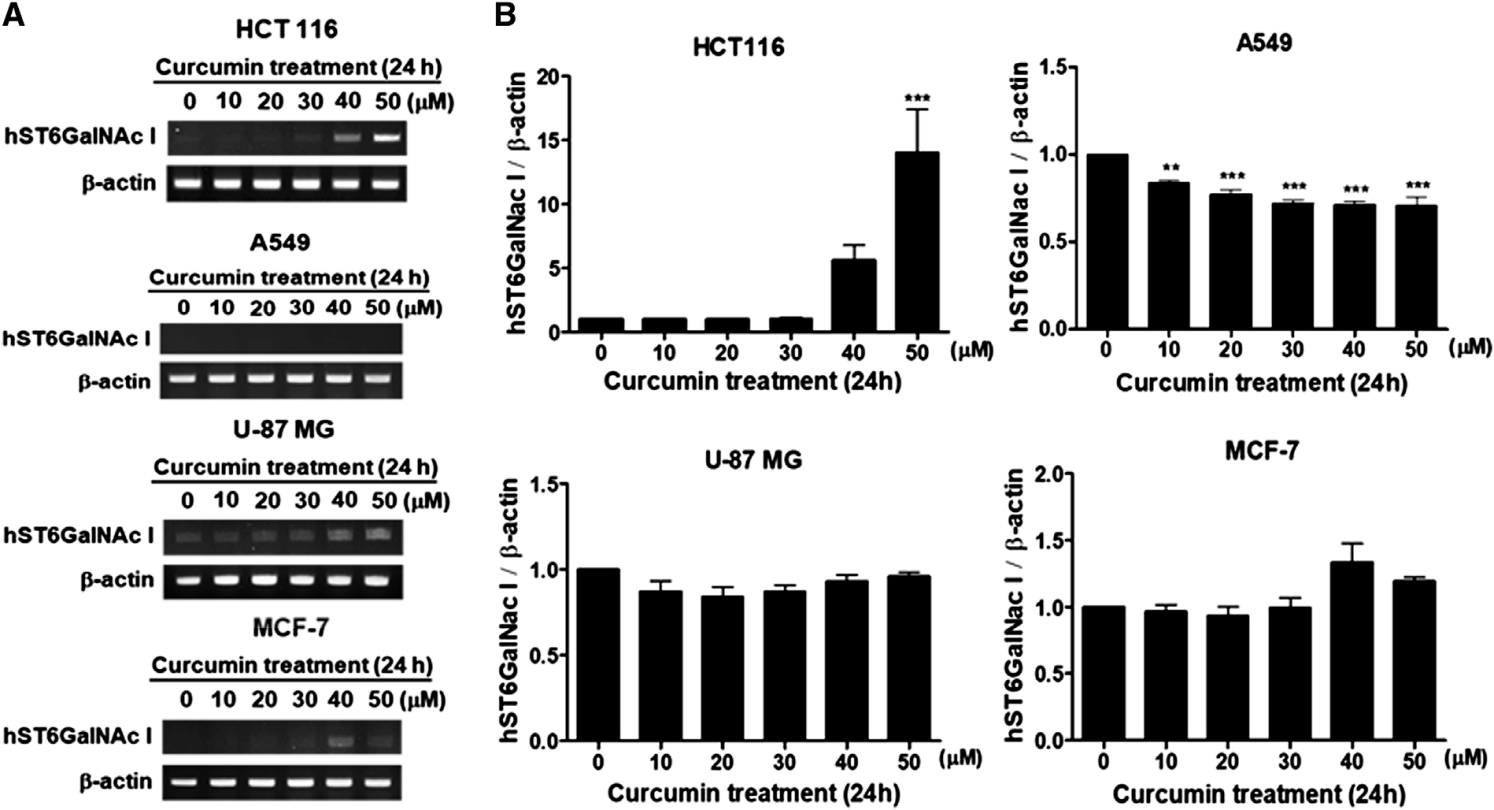
Effect of curcumin on hST6GalNAc I gene expression in HCT116, A549, U-87 MG and MCF-7 cells. After cells were treated for 24 h at different concentrations (0, 10, 20, 30, 40, 50 μM) of curcumin, mRNA levels of hST6GalNAc I were analyzed by RT-PCR using the extracted total RNAs **(A)**. β-actin mRNA was also analyzed as an internal standard. The densitometric intensity of hST6GalNAc I band was shown as percentages of the control (0 μM) in the panel below **(B)**. Results are presented as the mean ± SEM for three independent experiments. ***p < 0.001 compared with control cells untreated with curcumin.

In the previous studies, we showed that curcumin, a natural polyphenolic compound, increases gene expressions of ganglioside-specific human sialyltransferases, hST3Gal V and hST8Sia I, in human cancer cell lines (Lee at al., 2018 a, 2018b). In this study, we investigated the effect of curcumin on hST6GalNAc I gene expression in four kinds of human cancer cell lines (colon cancer cell HCT116, lung cancer cell line A549, glioblastoma cell line U-87 MG and breast cancer cell MCF-7). As shown in Figure 1, gene expression of hST6GalNAc I assessed by RT-PCR after treatment for 24 h with different concentration of curcumin was increased remarkably in HCT116 cells, which began to increase at 30 μM curcumin and showed about 14-fold enhancement at 50 μM curcumin compared to untreated control cells. However, a significant increase of hSTGalNAc I gene expression was not observed in A549, U-87 MG and MCF-7 cells treated with curcumin. This result indicates a cell type-specific hSTGalNAc I gene expression by curcumin.

### Curcumin elevates the expression of Sialyl-Tn antigen in HCT116 cells

**FIGURE 2:**
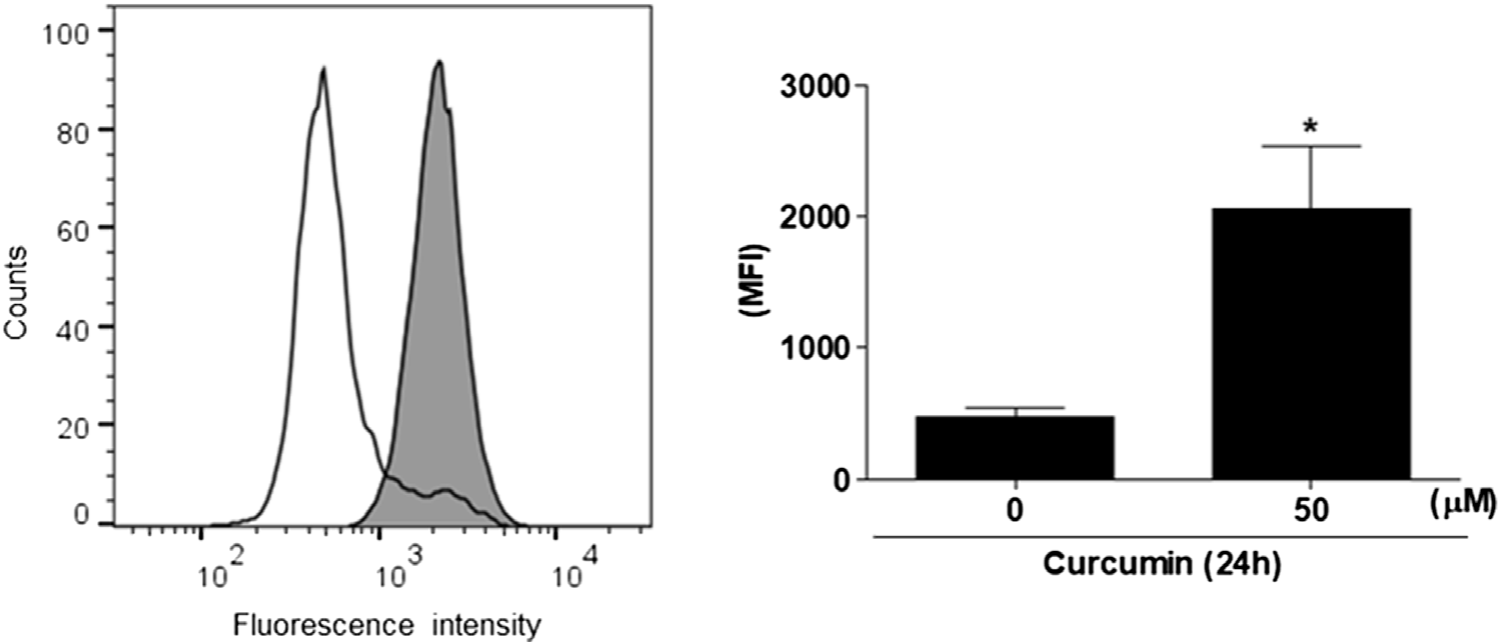
Flow cytometric analysis of sialyl Tn antigen using anti-sialyl Tn antibody in curcumin-treated HCT116 cells. After 50 μM curcumin treatment for 24 h, The cells (1 × 10^6^ cells) were analyzed by flow cytometry after incubation with anti-sialyl antibody in combination with the corresponding Alexa Fluor 488-conjugated goat anti-mouse IgG H&L secondary antibody. White and filled grey areas represent the histograms for cells treated with 0 and 50 μM curcumin, respectively. Results are presented as the means ± SEM of three independent experiments. *p < 0.05 compared with control cells untreated with curcumin.

To find out whether or not the augmented expression of hST6GalNAc I gene by curcumin causes the increase of sialyl-Tn antigen synthesized by hST6GalNAc I in HCT116 cells, the cellular expression level of hST6GalNAc I product was analyzed by flow cytometry using anti-sialyl Tn antibody and Alexa Fluor 488-conjugated goat anti-mouse IgG H&L secondary antibody. As shown in Figure 2, the binding of anti-sialyl Tn antibody to the HCT116 cell surfaces treated with 50 μM curcumin was increased a 4-fold compared to HCT116 cells untreated with curcumin.

### Cloning of the 5′-Flanking region of the hST6GalNAc I gene

**FIGURE 3:**
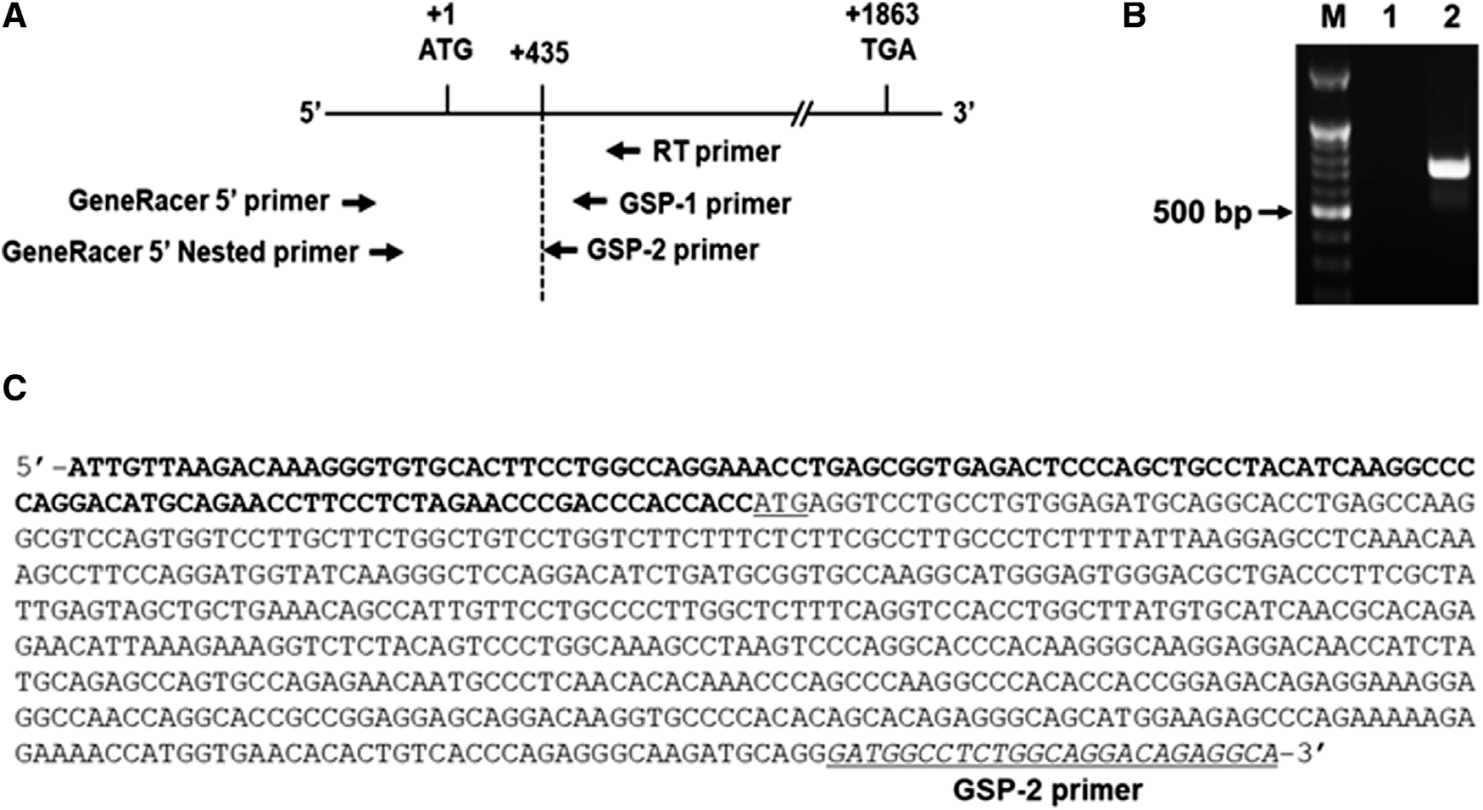
Identification of the transcription start site in the 5′-flanking region of hST6GalNAc I gene by 5′RACE-PCR in curcumin-induced HCT116 cells. Total RNA from HCT116 cells was prepared after 50 µM curcumin treatment for 24 h **(A)** RT reaction was performed using GSP RT primer and PCR was performed with Gene RACE primer and Gene specific primers with 5′-RACE strategy. **(B)** Agarose gel (1%) analysis of the 5′-RACE PCR product. Lane 1; GeneRacer 5′ primer and GSP1 primer, Lane 2; GeneRacer 5′ Nested primer and GSP2 primer. **(C)** Nucleotide sequences of the 5′-RACE PCR product (715 bp) amplified with GeneRacer 5′ Nested primer and GSP2 primer. 5′-UTR sequences are shown in bold letter and the initiation codon (ATG) is underlined.

As an initial step for cloning of the 5’ -flanking region including promoter region of the hST6GalNAc I gene, we conducted 5′-RACE using two gene-specific primers (GSP1 and 2) and total RNA from HCT116 cells treated with 50 μM curcumin for 24 h to determine the transcription start site of the hST6GalNAc I gene. As shown in Figure 3 about 0.7 kb product was obtained and sequenced after subcloning into pGEM-T Easy plasmid. From the sequence analysis of this product (714 bp), the transcription start site of the hST6GalNAc I corresponding to an adenosine nucleotide was 119 bp upstream of the translation start site (ATG). To identify the 5′-flanking region of the hST6GalNAc I gene by sequence overlap, the resulting 714 bp sequence was aligned and compared to the known nucleotide sequences of human genomic DNA in GenBank database of NCBI by using the BLASTN search. The analysis showed that hST6GalNAc I gene is located at chromosome 17, Chr17q25.1 (NCBI Reference Sequence: NC_000017.11). Using this sequence information, the proximal 5′-flanking region of hST6GalNAc I gene spanning 1974 bp was amplified by LA-PCR from human genomic DNA, cloned into the pGEM-T Easy vector, and sequenced.

### Functional analysis of the 5′-Flanking region of the hST6GalNAc I gene by curcumin in HCT116 cells

**FIGURE 4:**
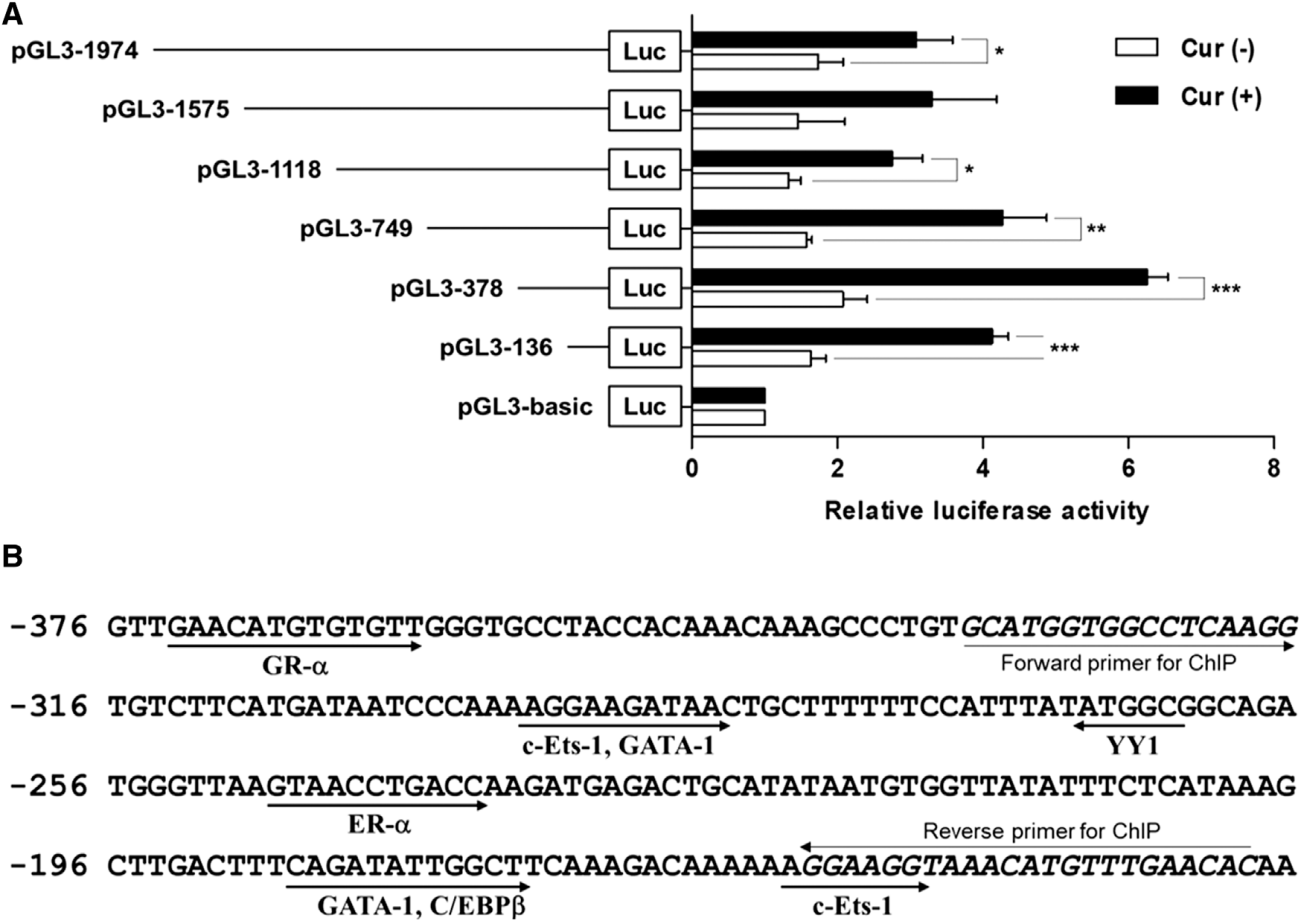
Analysis of the hST6GalNAc I promoter activity in HCT116 cells treated with curcumin. (A) Schematic diagrams show DNA constructs containing different 5′-deletion of the promoter region of the hST6GalNAc I ligated to the luciferase reporter gene. The pGL3-basic construct, which did not contain a promoter or an enhancer, was used as a negative control. Each construct was transfected into HCT116 cells, with pRL-TK co-transfected as an internal control. The transfected cells were incubated in the presence (solid bar) or absence (open bar) of 50 µM curcumin for 24 h. Relative firefly luciferase activity was measured using the Dual-Luciferase Reporter Assay System, and all firefly activity was normalized to the *Renilla* luciferase activity derived from pRL-TK. The values represent the means ± SEM of three independent experiments with triplicate measurements. *p < 0.05, **p < 0.01 and ***p < 0.001 compared with curcumin-untreated cells. **(B)** Nucleotide sequences of the promoter region from -376 to -136 and putative transcription factor binding sites (TFBS) are shown. TFBS were analyzed by using the ALGGEN-PROMO.v8.3 software with 100% matrix similarity rate. Forward and reverse sequences for ChIP are shown in *italic letters*.

To investigate whether the 5′-flanking region (1974 bp) of hST6GalNAc I includes its promoter region, the amplified 1974 bp fragment was subcloned into the reporter gene vector pGL3-Basic containing the firefly luciferase cDNA, resulting in pGL3-1974, and transfected into HCT116 cells followed by curcumin treatment. The promoter activity was assessed by luciferase reporter assay. As shown in Figure 4A, luciferase activity derived from the pGL3-1974 plasmid was significantly increased (1.8-fold) in curcumin-treated cells compared with untreated cells. Luciferase activity of control plasmid pGL3-basic was not affected by curcumin treatment, indicating that this region (1974 bp) has the curcumin-inducible promoter activity in HCT116 cells.

To further determine regions of the hST6GalNAc I promoter mediating the curcumin-inducible upregulation, we next constructed the serial 5′-deletion mutants of pGL3-1974 construct, transfected into HCT116 cells, and performed the promoter assay after curcumin treatment. As shown in Figure 4A, all constructs (pGL3-1575 to pGL3-136) showed the basal and curcumin-inducible promoter activities similar to pGL3-1974 construct. Among them, the pGL3-378 construct revealed the highest enhancement (3-fold increase) of promoter activity by curcumin stimulation compared with curcumin-untreated cells, whereas the pGL3-136 construct reduced promoter activity to 33% of the activity of the pGL3-378 construct. These results suggest that the region (242 bp) between -378 and -136 functions as a core region of hST6GalNAc I promoter mediating transcriptional activation by curcumin. Based on the result of RT-PCR shown in Figure 1, on the other hand, to address transcriptional activity of hST6GalNAc I in A549, U-87 MG and MCF-7 cells, four constructs (pGL3-1974 to pGL3-136) were transfected into these cells, and performed the promoter assay after curcumin treatment. No significant increase in curcumin-induced promoter activity was observed in these cells, indicating cell type-specific expression of hST6GalNAc I by curcumin (Supplementary Figure S1).

### Identification of curcumin-response element in the -378/-136 region of hST6GalNAc I promoter

**FIGURE 5:**
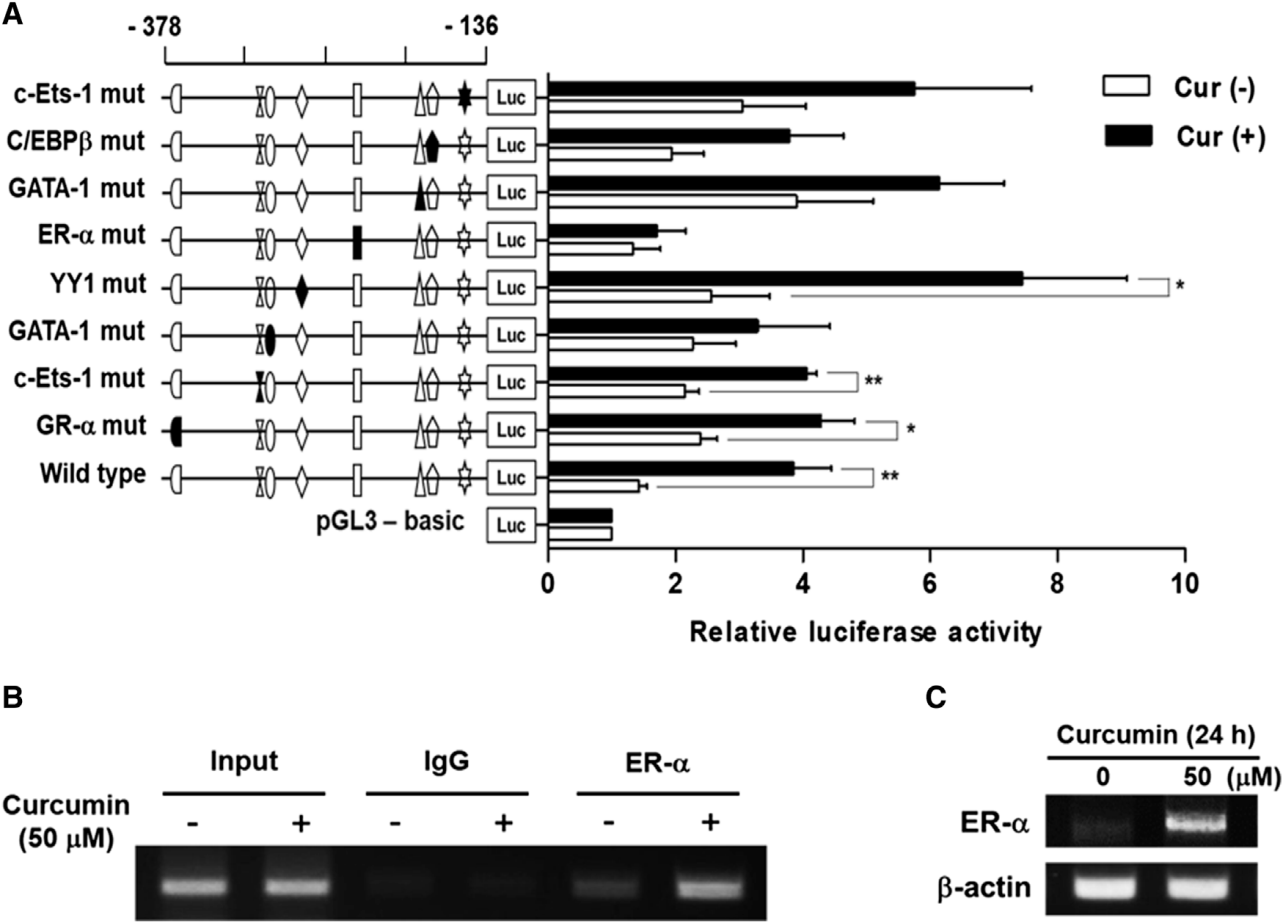
Effect of mutations in the putative transcription factor binding sites on hST6GalNAc I promoter activity and ChIP assay. **(A)** The mutation mark of promoter construction is indicated by closed form or opened form (wild-type). Each construct was transfected into HCT116 cells, with pRL-TK co-transfected as an internal control. The transfected cells were incubated in the presence (solid bar) or absence (open bar) of 50 µM curcumin for 24 h. Relative firefly luciferase activity was measured using the Dual-Luciferase Reporter Assay System, and all firefly activity was normalized to the *Renilla* luciferase activity derived from pRL-TK. The values represent the means ± SEM of three independent experiments with triplicate measurements. Symbols for transcription factor binding sites are displayed and solid symbols show the mutated sites. *p < 0.05, **p < 0.01 and ***p < 0.001 compared with curcumin-untreated cells. **(B)** ChIP assay was conducted in curcumin-treated HCT116 cells, or non-treated cells with input control (without antibody), nonspecific immunoglobulin (IgG), and ER-α antibody. The -333 and -138 region (195 bp) of the hST6GalNAc I promoter on immunoprecipitated chromatin obtained from HCT116 cells treated with or without curcumin was amplified by PCR. **(C)** After treatment of 50 μM curcumin for 24 h, mRNA levels of ER-α were analyzed by RT-PCR using the extracted total RNAs. β-actin mRNA was also analyzed as an internal standard.

To identify the transcription factors (TFs) modulating the curcumin-inducible hST6GalNAc I promoter activity in HCT116 cells, we searched for putative *cis* elements within the 242 bp sequence from −378 to −136 of the hST6GalNAc I gene using ALGGEN-PROMO.v8.3 online software. An *in silico* analysis of the 242 bp with 100% matrix similarity rate revealed several transcription factor response elements such as c-Ets-1, C/EBPβ, GATA-1, ER-α, YY1 and GR-α (Figure 4Β). To define which of these elements are functionally important for curcumin-induced transcription of the hST6GalNAc I in HCT 116 cells, eight mutant constructs [c-Ets-1(-160) mut, C/EBPβ mut, GATA-1(-185) mut, ER-α mut, YY1 mut, GATA-1(-290) mut, c-Ets-1(-294) mut and GR-α mut] were generated, transfected into HCT116 cells followed by curcumin treatment, and the promoter activity was assessed by luciferase reporter assay. As shown in Figure 5A, six mutants (c-Ets-1(-160) mut, C/EBPβ mut, GATA-1(-185) mut, GATA-1(-290) mut, c-Ets-1(-294) mut and GR-α mut) showed curcumin-inducible promoter activities almost similar to wild type construct (pGL3-378), whereas YY1 mut showed a 3-fold increase in promoter activity by curcumin treatment compared to the untreated control, suggesting that YYI acts as a repressor of hST6GalNAc I transcription. On the other hand, the promoter activity of estrogen receptor-α (ER-α) mut was markedly decreased by curcumin stimulation compared to the wild type construct. This result indicates that ER-α binding site at position −248 to −238 is crucial for the curcumin-mediated upregulation of hST6GalNAc I gene in HCT116 cells. To further verify whether ER-α could bind to its binding site in the hST6GalNAc I promoter, the *in vivo* interaction of ER-α with promoter region between -248 and -238 of hST6GalNAc I gene in curcumin-treated HCT116 cells was evaluated by ChIP assay. As shown Figure 5B, PCR amplification signal using chromatin DNA precipitated with ER-α antibody was significantly stronger in curcumin-treated cells than curcumin-untreated cells, whereas amplification of hST6GalNAc I promoter sequence was not detected in the control IgG immunoprecipitate. In addition, a marked increase of ER-α gene expression in HCT116 cells by curcumin treatment was confirmed by RT-PCR (Figure 5C). These results suggest that curcumin stimulation in HCT116 cells triggers the direct binding of ER-α to its binding site located in the hST6GalNAc I promoter region, leading to the upregulation of hST6GalNAc I gene expression.

### Curcumin-induced transcription activity of hST6GalNAc I gene is associated with PKC/ERKs pathway in HCT116 cells

**FIGURE 6:**
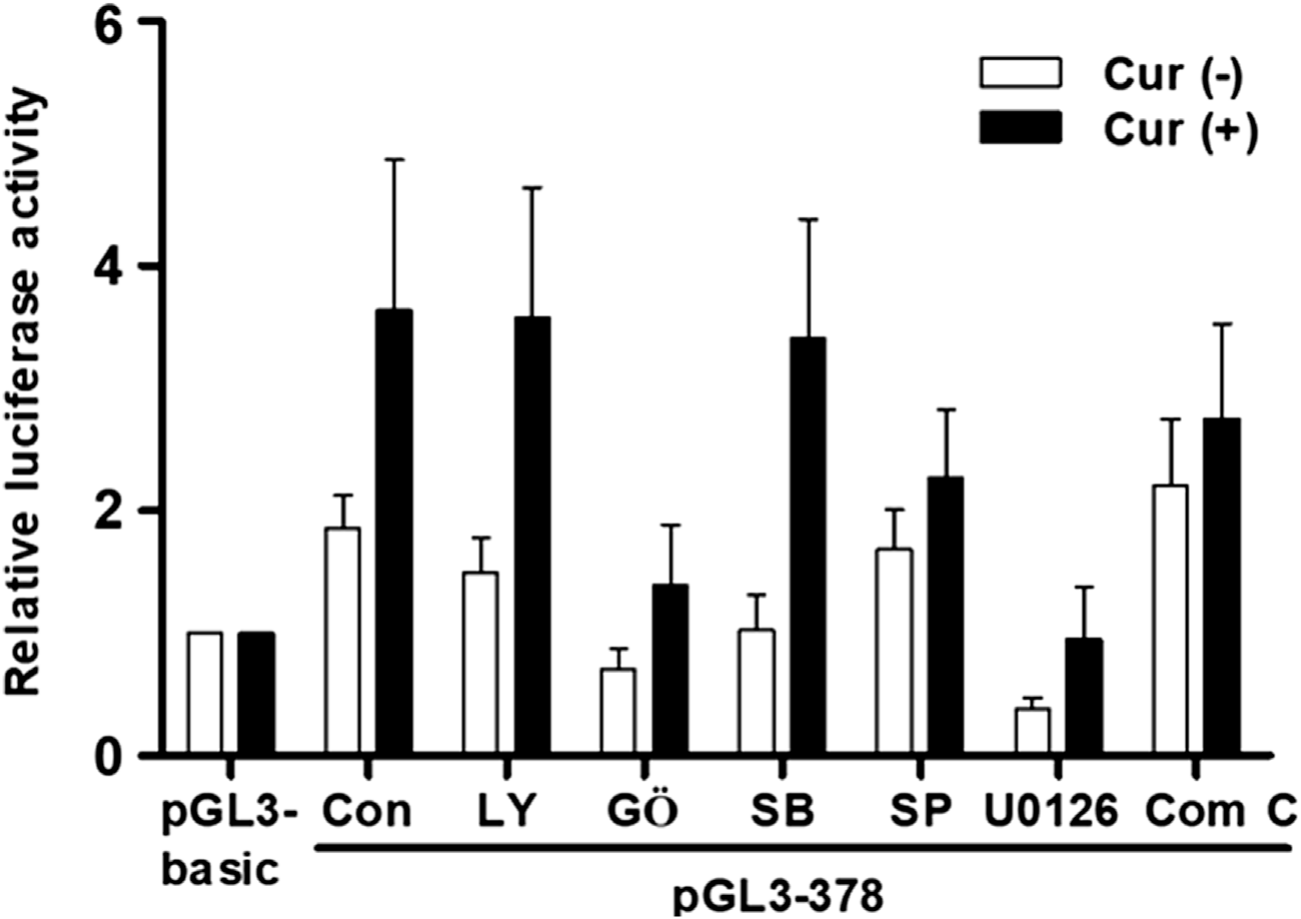
Effect of curcumin on signaling pathway controlling transcription of hST6GalNAc I in HCT116 cells. The pGL3-378 (positive control), pGL3-basic (negative control) and pRL-TK (internal control) were co-transfected into HCT116 cells. Transfected cells were incubated in the presence (solid bar) and absence (open bar) of 50 μM curcumin with LY294002 (10 μM), U0126 (10 μM), SP600125 (10 μM), GŐ6983 (100 nM), and Compound C (10 μM) inhibitors for 24 h. Relative luciferase activity was normalized with the *Renilla* luciferase activity derived from pRL-TK. Data represent mean ± SEM for three independent experiments with triplicate measurements.

We have recently found that curcumin-induced transcription activities of hST3Gal V and hST8Sia I genes in HCT116 and A549 cells, respectively, are mediated by AMPK signaling pathway (Lee et al., 2018a; 2018b). Therefore, we explored the signal transduction pathway modulating curcumin-mediated transcription activity of hST6GalNAc I gene in HCT116 cells. As shown in Figure 6, promoter activity of pGL3-378 as a control was increased in curcumin-stimulated HCT116 cells compared to unstimulated HCT116 cells. This characteristic was not significantly influenced by LY294002 (PI3K/AKT inhibitor) and SB203580 (p38 MAPK inhibitor), whereas SP600125 (JNK inhibitor) and compound C (AMPK inhibitor) had a slight effect on promoter activity of pGL3-378 by curcumin. However, GŐ6983 (PKC inhibitor) and U0126 (MEK/ERK inhibitor) treatments resulted in a marked reduction of pGL3-378 activity in curcumin-induced HCT 116. These results suggest that curcumin-mediated transcription activity of hST6GalNAc I gene in HCT116 cells is regulated by PKC/ERKs signaling pathway.

## Discussion

Here we firstly demonstrated that gene expression of human *N*-acetylgalactosamine-α2,6-sialyltransferase (hST6GalNAc I) catalyzing the synthesis of sialyl-Tn (sTn) antigen was upregulated by curcumin in HCT116 cells. In parallel with curcumin-induced enhancement of hST6GalNAc I gene expression, we verified the increase of sTn antigen structure by curcumin in HCT116 cells, as proven by FACS analysis using anti-sialyl Tn antibody. However, a significant increase of hSTGalNAc I gene expression by curcumin was not observed in A549, U-87 MG and MCF-7 cells, indicating a cell type-specific hSTGalNAc I gene expression by curcumin. Similarly, previous studies showed that most breast cancer cell lines, such as MCF-7, MDA-MB-231 and T47D (Julien et al., 2001, 2006; Sewell et al., 2006), and lung cancer cell line, including A549 (Lakshmanan et al., 2021), do not express hST6GalNAc I mRNA transcript or sTn antigen.

It is well known that gene expression of sialyltransferases is regulated at the transcriptional level in a cell type-, tissue-, and stage-specific manner through alternative splicing and alternative promoter usage (Harduin-Lepers et al., 2001; Dorsett et al., 2021).

Munkley et al. (2015) reported that expressions of hST6GalNAc1 gene and sTn is induced by androgen in human prostate cancer cell line LNCaP, and furthermore, using ChIP-qPCR they demonstrated the androgen-inducible hST6GalNAc1 gene expression in LNCaP cells by direct binding of androgen to an androgen receptor-binding site in close proximity to the hST6GalNAc1 promoter (Munkley et al., 2015). Pinto et al. (2015) also reported the time-dependent increase of hST6GalNAc I gene expression with an enhancement of the homeobox transcription factor CDX2 protein expression during differentiation of Caco-2 intestinal cell line. In addition, they demonstrated by ChIP analysis that CDX2 binds to the regulatory region of hST8GalNAc I gene in Caco-2 cells and human intestinal metaplasia, and luciferase reporter assay showed that CDX2 transactivates the regulatory region of hST8GalNAc I gene in human gastric carcinoma cell lines AGS and MKN45 (Pinto et al., 2015). In this study, we showed the cell type-specific expression of the hST6GalNAc I gene in response to curcumin in HCT116 cells and demonstrated by ChIP analysis and site-directed mutagenesis that hST6GalNAc I gene expression in response to curcumin stimulation in HCT116 cells is regulated by estrogen receptor α (ER-α) binding site in its promoter region. In line with the previous studies (Munkley et al., 2015; Pinto et al., 2015), our result in this study suggests that hST6GalNAc I gene expression is differentially controlled by cell type-specific promoter and/or cell type-specific transcriptiona factors in response to specific stimuli and different physiological signals, as in the case of hST6Gal I and hST3Gal IV (Taniguchi et al., 2003; Colomb et al., 2017; Dorsett et al., 2021).

An accurate determination of transcriptional start site (TSS) of an mRNA molecule is essential for identifying the regulatory region such as promoter that controls gene expression in an organism and the most reliable way to identify a TSS is to determine a nucleotide to which a 5′-cap structure is attached in the mRNA (Kapranov, 2009). In this study, we first defined TSS of hST6GalNAc I gene by 5′-RACE which is most widely used to determine transcription start site (Scotto-Lavino et al., 2006), using cDNA derived from curcumin-treated HCT 116 cells. Based on this result, curcumin-inducible promoter region of hST6GalNAc I was cloned and characterized in HCT 116 cells.

Here we revealed by deletion analysis of the hST6GalNAc I promoter that the promoter region spanning nucleotides -378 to -136 is critical for curcumin-inducible transcriptional activity in HCT116 cells. Furthermore, we clarified that promoter activity of hST6GalNAc I was not increased significantly in A549, U-87 MG and MCF-7 cells, which do not express the hST6Gal NAc I gene, demonstrating cell type-specific expression of the hST6GalNAc I gene in HCT116 cells.

Several transcription factor response elements such as c-Ets-1, C/EBPβ, GATA-1, ER-α, YY1 and GR-α were identified in the region of nucleotides −378 to −136 in the hST6GalNAc I promoter. Among these elements, we demonstrated that estrogen receptor-α (ER-α) is the key transcription factor for curcumin-induced transcriptional activation of hST6GalNAc I gene in HCT116 cells, as evidenced by site-directed mutagenesis and ChIP assay.

ER-α is one of the estrogen ligand-activated transcription factors that bind to an estrogen-responsive element (ERE) in target gene promoters, leading to modulate their transcription (Reid et al., 2002; O’Donnell et al., 2005; Arnal et al., 2017). Especially ER-α is well known as the primary transcription factor that mediates estrogen signaling in breast cancer biology (Sotiriou and Pusztai, 2009; Miyoshi et al., 2010; Siersbæk et al., 2018; Rusidzé et al., 2021). ER-α was highly expressed in human breast and ovarian cancer cell lines (O’Donnell et al., 2005; Sotiriou and Pusztai, 2009; Miyoshi et al., 2010; Siersbæk et al., 2018), while its mRNA was not detected in human colon cancer cell lines, such as HCT8 and HCT116 (Fiorelli et al., 1999). Moreover, regulation of ER-α gene expression has been well documented in human breast cancer cell lines but to date little is known about in human colon cancer cell lines. In this study, we firstly demonstrated that ER-α gene expression in HCT116 cells was remarkably upregulated by curcumin.

In the previous study, we showed that transcriptional regulation of human sialyltransferase hST3Gal V by curcumin in HCT116 cells was modulated through AMPK signaling pathway (Lee et al., 2018a). We also reported that transcriptional activation of human sialyltransferase hST8Sia I by curcumin in A549 cells was controlled via AMPK signaling pathway (Lee et al., 2018b). Contrary to these reports, the present result demonstrated that curcumin-induced hST6GalNAc I gene expression in HCT116 cells is regulated through PKC/ERKs signal pathway.

Although the precise mechanisms involved in the curcumin-mediated gene expression and transcription activity of ER-α in HCT116 cells, including the mechanism of ER-α activation by PKC/ERKs signal pathway, leading to a transcriptional activation of the hST6GalNAc I gene are unknown, we have for the first time demonstrated in this study that the PKC/ERKs-dependent ER-α activation upregulates the hST6GalNAc I gene expression in curcumin-stimulated HCT116 cells.

In conclusion, we have for the first time determined the TSS of hSTGalNAc I gene and characterized its 5′-flanking region. We found curcumin-inducibe promoter region for maximal transcriptional activity in HCT116 cells. We identified ER-α binding site within the hST6GalNAc I promoter to be crucial for curcumin-inducible promoter activity in HCT116 cells.

## Data Availability

The datasets presented in this study can be found in online repositories. The names of the repository/repositories and accession number(s) can be found in the article/Supplementary Material.
