# Supplementary figures and images for: Curcumin-mediated transcriptional regulation of human N-acetylgalactosamine-α2,6-sialyltransferase which synthesizes sialyl-Tn antigen in HCT116 human colon cancer cells
Source: Front Mol Biosci. 2022 Sep 12;9:985648. doi: 10.3389/fmolb.2022.985648 (PMC9510914; doi:10.3389/fmolb.2022.985648)

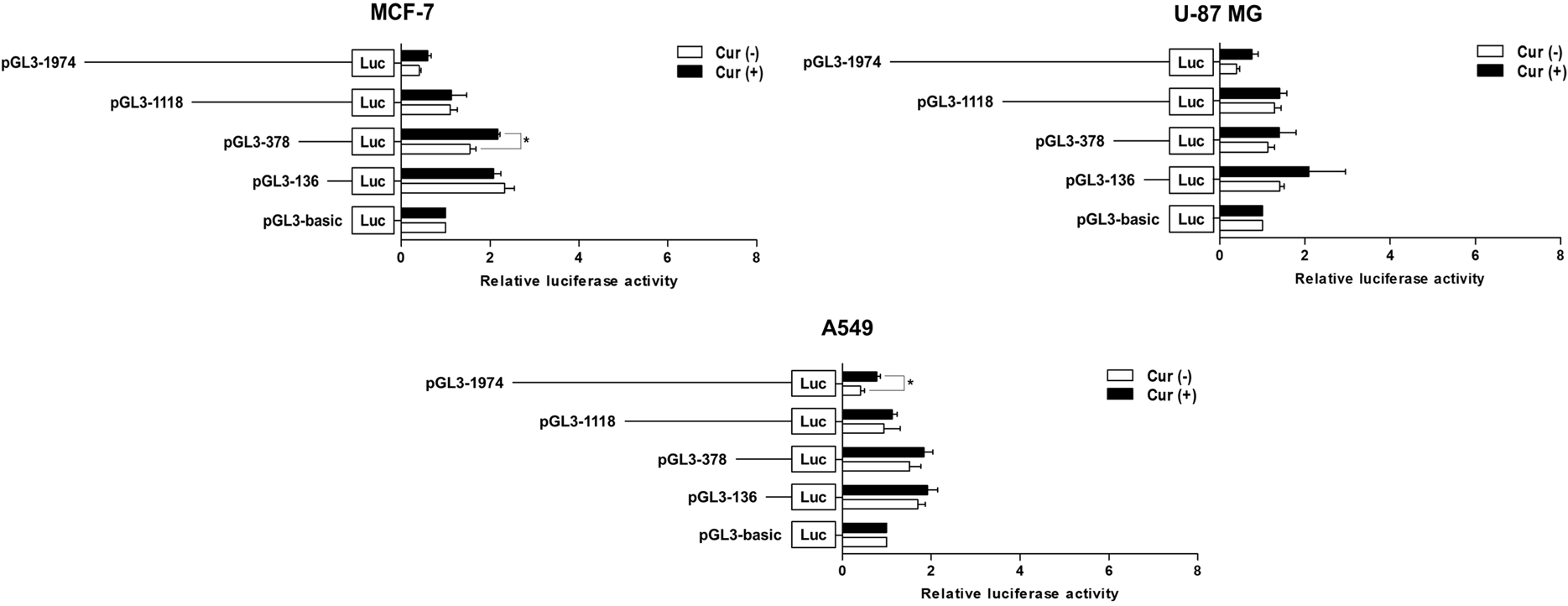

Supplement: Supplementary file 1 [file Image1.TIF]
